# Peptide Analysis by Soft X‑ray Atmospheric Pressure Photoionization Mass Spectrometry

**DOI:** 10.1021/jasms.5c00037

**Published:** 2025-05-19

**Authors:** Simona Sedláčková, Juha-Pekka Hieta, Miroslava Blechová, Josef Cvačka

**Affiliations:** † Institute of Organic Chemistry and Biochemistry of the Czech Academy of Sciences, Flemingovo nám. 2, CZ-166 10 Prague 6, Czech Republic; ‡ Department of Analytical Chemistry, Faculty of Science, Charles University in Prague, Hlavova 2030/8, CZ-128 43 Prague 2, Czech Republic; § Drug Research Program and Division of Pharmaceutical Chemistry and Technology, Faculty of Pharmacy, University of Helsinki, P.O. Box 56, FI-00014 Helsinki, Finland

## Abstract

Bottom-up proteomics typically involves enzymatic digestion of proteins, generating a complex peptide mixture. These peptides are separated using reversed-phase ultrahigh-performance liquid chromatography (UHPLC) and analyzed using electrospray ionization (ESI) tandem mass spectrometry (MS/MS) in positive ion mode. Despite its widespread use, this approach has limitations, particularly in ionizing highly acidic or hydrophobic peptides and detecting certain post-translational modifications (PTMs). To overcome these challenges, alternative ionization methods, such as vacuum ultraviolet (VUV) atmospheric pressure photoionization (APPI), have been explored. In this study, we propose peptide analysis using a novel prototype APPI source employing soft X-ray photons. Soft X-ray photons possess orders of magnitude higher energy than VUV photons, enabling additional ionization pathways. Here, we present peptide ionization data using soft X-ray and VUV APPI in both positive and negative ion modes. Notably, soft X-ray photons exhibited a remarkable capacity to generate deprotonated peptides and hydrogen-deficient peptide radical anions ([M – 2H]^•–^), outperforming conventional VUV photons. Furthermore, collision-induced dissociation (CID) of [M – 2H]^•–^ provided unique structural insight, facilitating PTM characterization. Our findings emphasize the significant potential of soft X-ray APPI in advancing peptide analysis and highlight the utility of negative ion mode for proteomic applications.

## Graphical abstract

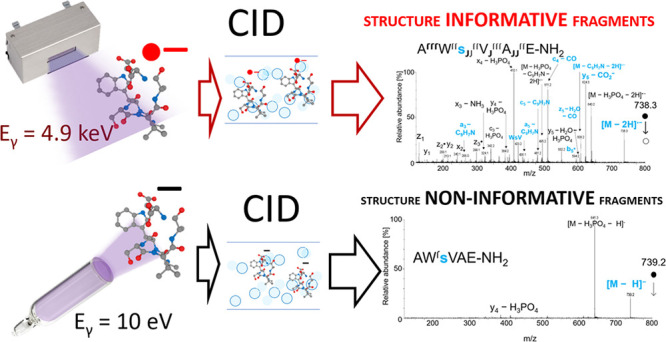

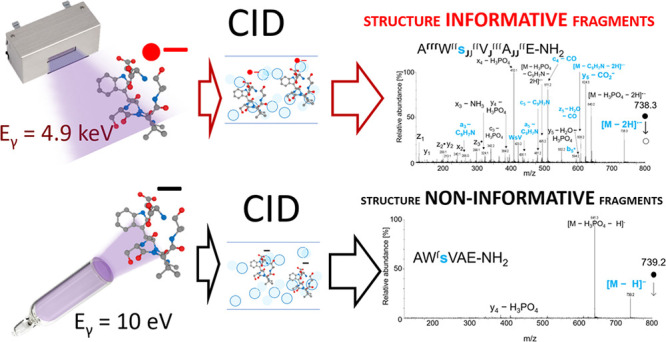

## Introduction

Bottom-up proteomics is a widely used mass spectrometry approach in protein research. The standard workflow involves protein digestion with trypsin, followed by peptide separation using reversed-phase ultrahigh-performance liquid chromatography (UHPLC). The peptides are then ionized in electrospray and analyzed by tandem mass spectrometry (MS/MS) by collision-induced dissociation (CID). Automated database search engines are employed to interpret the data.

Operating the ion source in positive ion mode offers distinct advantages for tryptic digests, as peptides containing C-terminal basic amino acids are efficiently protonated, enhancing detection sensitivity. Trypsin specifically targets arginine and lysine residues; however, the number of these cleavage sites may be limited, particularly in hydrophobic proteins such as membrane proteins.^1^ Trypsin sensitivity can be additionally reduced by post-translational modifications (PTMs) of arginine or lysine residues or by adjacent acidic amino acids at cleavage sites.^2^ Such proteins include e. g. human adenovirus proteome,^3^ HeLa proteome,^4^ human heart tissue,^5^ or cerebrospinal fluid proteins.^6^ These proteins are more effectively cleaved by proteases that target peptide bonds at the C-terminal side of hydrophobic or acidic amino acids. Peptides with acidic C-termini have more sites for deprotonation, making them more easily detectable in negative ion mode.^7,8^ Additionally, PTMs such as phosphorylation,^9^ sulfation,^10^ and sialylation^11^ can enhance peptide acidity, further improving detection efficiency in negative ion mode. One major challenge in integrating negative ion mode into standard bottom-up proteomics is the limited understanding of the fragmentation behavior of deprotonated peptides. Although several studies have investigated the fragmentation pathways,^12–23^ mass spectrometry of negatively charged peptide ions remains insufficiently explored. Unlike positive ion mode, where b- and y-ions dominate, the primary fragment ions in negative ion mode are c- and z-ions. In addition to backbone cleavages, deprotonated peptides frequently exhibit neutral losses of small molecules such as formaldehyde, acetaldehyde, ammonia, and water in CID spectra. Meanwhile, radical peptide anions follow distinct fragmentation pathways that preserve PTMs, allowing for deeper structural insights.^24–28^ Software tools for interpreting fragmentation spectra of peptides are less developed for negative than for positive ion mode. However, ongoing research aims to better characterize negative ion fragmentation pathways and improve software solutions to expand their applications in proteomics. In some cases, negative ion mode provides superior structural information compared to positive ion mode, particularly for smaller peptides (5–10 amino acids),^14^ as well as for identifying disulfide bonds,^29^ sulfonation,^10^ and phosphorylation.^25^

Electrospray ionization (ESI) is a fundamental and powerful technique in proteomics; however, it has notable limitations, including susceptibility to matrix effects in biological samples^30^ and challenges in ionizing certain classes of peptides.^31^ A fundamentally different ionization method, atmospheric pressure photoionization (APPI), can help overcome these limitations.^32,33^ APPI is particularly effective for hydrophobic peptides and is significantly less influenced by matrix effects than ESI,^34^ making it useful for identifying membrane proteins.^31,35^ Commercial APPI sources operate optimally at relatively high flow rates (hundreds to thousands of microliters per minute); however, ongoing research aims to enhance detection sensitivity at low flow rates in the microliters-per-minute range.^36,37^ In APPI, the photon source is typically a krypton discharge lamp that emits photons with wavelengths of 116.5 nm (10.6 eV) and 123.6 nm (10 eV). The photon energy is often below the ionization energies (IE) of common LC solvents and atmospheric gases, minimizing unwanted background ionization. Dopants with low IE are used to enhance the efficient ionization of analytes. Synchrotron radiation offers a versatile alternative, allowing the selection of photons across a broad energy range. APPI utilizing synchrotron radiation with photon energies in the 4–20 eV has been shown to efficiently ionize peptides and generate structure-informative, odd-electron peptide fragments.^38^ This fragmentation is believed to occur via electron transfer to multiply charged peptides, leading to N–Cα or Cα-C peptide backbone cleavage through an electron-mediated dissociation mechanism. Similar fragments can be obtained using electron-based techniques^26,38–41^ and photon activation methods.^42–44^ Additionally, odd-electron peptide fragments can be generated via CID, though peptide modification is required.^25,45,46^

Recently, a novel in-house-developed soft X-ray APPI source has been introduced.^47^ The primary difference between soft X-ray and vacuum-ultraviolet (VUV) APPI lies in the photon energy. Soft X-ray APPI operates at a tube voltage of 4.9 kV, enabling the emission of photons with energies up to approximately 4.9 keV. This energy is orders of magnitude higher than that of photons emitted by krypton VUV lamps. However, the exact emission spectrum of the soft X-ray ion source remains unspecified. While low-energy photons induce electronic excitations and valence electron ejection, the highly energetic soft X-ray photons can interact with and eject core electrons deeply bound in inner shells. The absorption of these high-energy photons can lead to electron ejection, initiating further ionization processes. Additional electrons can be emitted by metal surfaces inside the ion source. Consequently, APPI spectra reflect the complex interactions involving analytes, solvents, and gases in both the gas phase and on surfaces.

This study evaluates the potential of the newly introduced soft X-ray APPI for peptide analysis. Experiments were conducted using 21 peptide standards, ranging from four to nine amino acids with diverse terminal residues. Some peptides were modified by phosphorylation, lipidation, or amidation. Compared to VUV APPI, soft X-ray APPI produced significantly more abundant signals of deprotonated molecules in negative ion mode. Additionally, radical-driven ionization and fragmentation pathways generated hydrogen-deficient radical anions, highlighting the potential of soft X-ray APPI as a powerful tool for peptide structural analysis, including PTMs.

## Experimental Section

### Reagents and Materials

Methanol and acetonitrile (OPTIMA LC/MS) were obtained from Fisher Chemical (Waltham, MA, USA). Fmoc Arg­(Pbf) WANG resin, Fmoc Lys­(Boc) WANG resin, Fmoc Phe WANG, and Fmoc Glu­(OtBu) WANG, along with diisopropyl carbodiimide (DIC) and oxyma-ethylcyanohydroxyiminoacetate (Oxyma), used for peptide standards synthesis, were purchased from Iris Biotech (Marktredwitz, Germany). Dimethylformamide (DMF, HPLC grade) was acquired from VWR International (Stribrna Skalice, Czech Republic). Triisopropyl silane (TIS, 98%) was sourced from Acros Organics B.V.B.A. (Geel, Belgium). *Tert*-butyl methyl ether (99.8%), trifluoroacetic acid (TFA) for HPLC (>99.9%), and acetic anhydride (97%) were obtained from Sigma-Aldrich (St. Louise, WA, USA). Ultrapure water was produced using a Milli-Q water purification system (Millipore, Molsheim, France).

### Peptide Standards

Peptide standards SLGF, VASLR, VASLE, VASLF, AWSVAR, AWSVAF, AWSVAE, VLASSAR, VLASSAE, VLASSAF, AEFVEVTK, AEFVEVTE, AEFVEVTF, QTALVELLK, QTALVELLE, QTALVELLEF, GVSGSK-NH_2_, AWSVAE-NH_2_, AWSVAF-NH_2_, *N*-myristoyl GVSGSK-NH_2,_ and *N*-palmitoyl GVSGSK-NH_2_ were synthesized in-house (see Text S1 for details). Peptide acetylation was carried out by reacting the peptides with an acetylation reagent (acetic anhydride and methanol, 1:3 v/v) at 25 °C for 1 h, following a previously described protocol.^32^

Stock solutions of peptide standards (1 mg/mL) for positive ion mode analysis were prepared in acetonitrile/water (1:1 v/v) containing 0.1% TFA. For MS analysis in negative ion mode, peptides were suspended in Milli-Q water (1 mg/mL) and subjected to ultrasonic treatment for up to 60 min, followed by incubation at 65 °C for 30 min. Undissolved residues were removed using a 0.2 μm syringe filter (13 mm glass fiber hydrophilic propylene (GHP) (Pall Life Sciences, Portsmouth, UK). Peptide stock solutions were stored at −80 °C. Working solutions were prepared prior to MS analysis by diluting stock solutions 60-fold with Milli-Q water for both ion modes.

### Mass Spectrometry

The experiments were conducted using a Xevo quadrupole time-of-flight mass spectrometer (Q-TOF-MS) (Waters Corp., Manchester, UK) coupled with an ACQUITY UPLC system (Waters Corp., Milford, MA, USA). Peptide solutions were introduced into the mass spectrometer via a syringe pump (Pump 11 Elite, Harvard apparatus; Harvard Bioscience Inc., Holliston, MA, USA) at a flow rate of 10 μL/min and mixed with acetonitrile at a flow rate of (100–200 μL/min). In negative ion mode, the flow rate was optimized to maximize the abundance of peptide radical anions, with an optimal Milli-Q water flow rate of 40 μL/min). The flow rate optimization details in negative ion mode are provided in Text S2 and Figure S1.

Mass spectra were acquired at a data acquisition frequency of 2 Hz. Tuning parameters were optimized manually. The sampling cone and extraction voltages were set to 30.0 and 1.0 V, respectively. The source temperature was maintained at 130 °C, while the probe temperature was set to the maximum of 650 °C. The cone and desolvation gas flow rates were 1 L/h and 200 or 600 L/h, respectively. The ion isolation was performed in Q1, and CID was carried out in the collision cell. The optimal normalized collision energy (NCE) values for peptide ions ranged from 26% to 50%. The ion isolation window was optimized individually by adjusting the low and high mass resolution, aperture 1, and prefilter settings. Ion energy, which influences the degree of fragmentation and ion transfer efficiency, was set to 0 V.

The soft X-ray APPI system was built in-house to the commercial Waters Zspray Nanoflow ion source frame.^47^ The nano-ESI probe was replaced with a Zspray atmospheric pressure chemical ionization (APCI) nebulizer. A soft X-ray source (PhotoIonBar L12536; Hamamatsu Photonics) or a VUV lamp (PKR 100; Heraeus Noblelight) was placed by built-in XY stages in front of the MS inlet. The VUV lamp emitted photons at 10.0 and 10.6 eV, while the soft X-ray source emitted 4.9 keV photons, powered by a separate controller (C12537; Hamamatsu). Only one emitter was used at a time. To ensure safety, harmful soft X-ray photons were blocked using aluminum foil, and the operator maintained a safety distance of two meters from the soft X-ray ion source.

Ultraviolet photodissociation (UVPD) spectra were acquired using an Orbitrap IQ-X Tribrid (Thermo Scientific, Waltham, MA, USA) equipped with a TriVersa NanoMate robotic chip-based nanoESI system (Advion, Inc., Ithaca, NY, USA), controlled by Chipsoft 8.3.1 software. The ionization spray voltage was set to 1.45 kV, and the nitrogen gas pressure was maintained at 0.4 psi. Sample solutions were loaded from 96-well plates, and the sample aliquots (5–10 μL) were infused directly into the mass spectrometer.

### Data Interpretation

The MS data obtained in VUV and soft X-ray APPI were processed in Mass Lynx (version 4.2, Waters Corp., Milford, MA, USA). Calculations of the peptides’ isotopic contributions in negative ion mode are specified in Text S3. The UVPD MS/MS spectra were evaluated in Xcalibur (version 4.1, Thermo Fisher Scientific, Waltham, MA, USA).

### Peptide Fragment Notation

A previously established notation to describe peptide fragments of both charge polarities was adopted.^48^ Fragments from the N-terminus are labeled as a, b, and c, while those from the C-terminus are labeled as x, y, and z. The a- and x-fragments result from Cα-C bond cleavage, b- and y-ions from C–N bond cleavage, and c- and z-ions from N–Cα bond cleavage. In contrast to common Biemann notation,^49^ the charge, radical, and hydrogens gained or lost are explicitly annotated.

## Results and Discussion

Peptide standards were analyzed using soft X-ray and VUV APPI in both positive and negative ion modes to compare detection sensitivity and peptide fragmentation across the two ion sources.

### APPI Spectra of Peptides in Positive Ion Mode

The detection of peptide standards in positive ion mode using soft X-ray and VUV APPI was investigated, both with and without the photon source activated. Initially, the ZSpray source was operated with the photon source turned off, revealing abundant peptide adducts and fragments, likely resulting from thermospray-like ionization. These ions persisted in the spectrum even after the photon source was turned on (Figure 1). The thermospray phenomenon observed during the APPI process originated from a heated pneumatic sprayer and has been extensively reviewed by Robb and Blades.^50^

**Figure 1:**
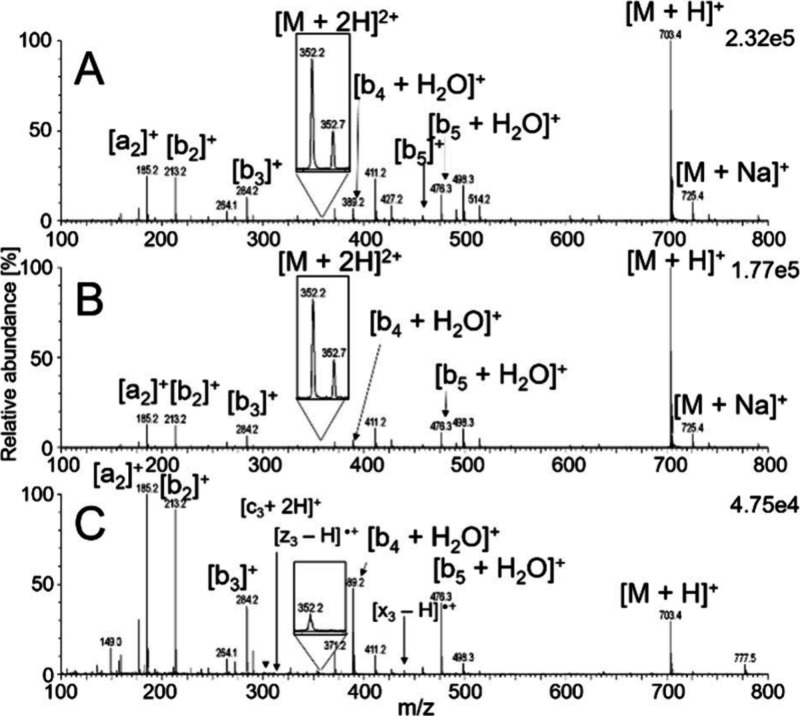
Full-scan mass spectra of VLASSAR obtained using soft X-ray APPI with the photon source turned off (A), VUV APPI (B), and soft X-ray APPI (C). The inset is magnified approximately 30-fold. The Waters ZSpray was operated in positive ion mode with a probe temperature of 650 °C. The peptide was dissolved in acetonitrile–water (9:1, v/v) and infused from a syringe (10 μL/min) into a 200 μL/min acetonitrile flow.

The full-scan mass spectra acquired without photoirradiation primarily exhibited singly charged protonated molecules and sodium adducts. Low-abundance signals of doubly charged peptides were detected for peptides with lysine and arginine at the C-terminus. Ionization processes within the source led to the neutral loss of water or ammonia, along with the formation of b-, y-, and low-abundance a-ions. The presence of a-ions was attributed to the neutral loss of carbon monoxide from b-ions^51,52^ (Figure 1A). All these ions remained in the spectra after photon activation (Figures 1B, C); however, the abundances of [M + 2H]^2+^, [M + H]^+^, and [M + Na]^+^ decreased for most peptides. This reduction was accompanied by increased peptide fragmentation, particularly in soft X-ray APPI (Figure 1C).

In the full-scan soft X-ray APPI spectra, peptides with lysine or arginine at their C-terminus, along with some others, exhibited low-abundance fragments corresponding to N–Cα and Cα-C bond cleavages. The presence of low abundant [z_3_ – H]^•+^, [x_3_ – H]^•+^ (Figure 1C) indicate the involvement of radical-driven fragmentation pathways. Notably, N–Cα and Cα-C bond cleavages were also observed in the full-scan spectrum of AWsVAE-NH_2_, an amidated peptide phosphorylated at serine (Figure 2A). The spectrum displayed numerous a-, x-, and z-ions, as well as internal fragments. In contrast, CID MS/MS of [M + H]^+^ primarily produced b- and y-ion series, accompanied by abundant neutral losses of H_3_PO_4_ and NH_3_ (Figure 2B). The fragmentation patterns observed in soft X-ray APPI suggest that, in addition to vibrational energy-driven dissociation, radical-based fragmentation or direct dissociation processes also contribute.

**Figure 2:**
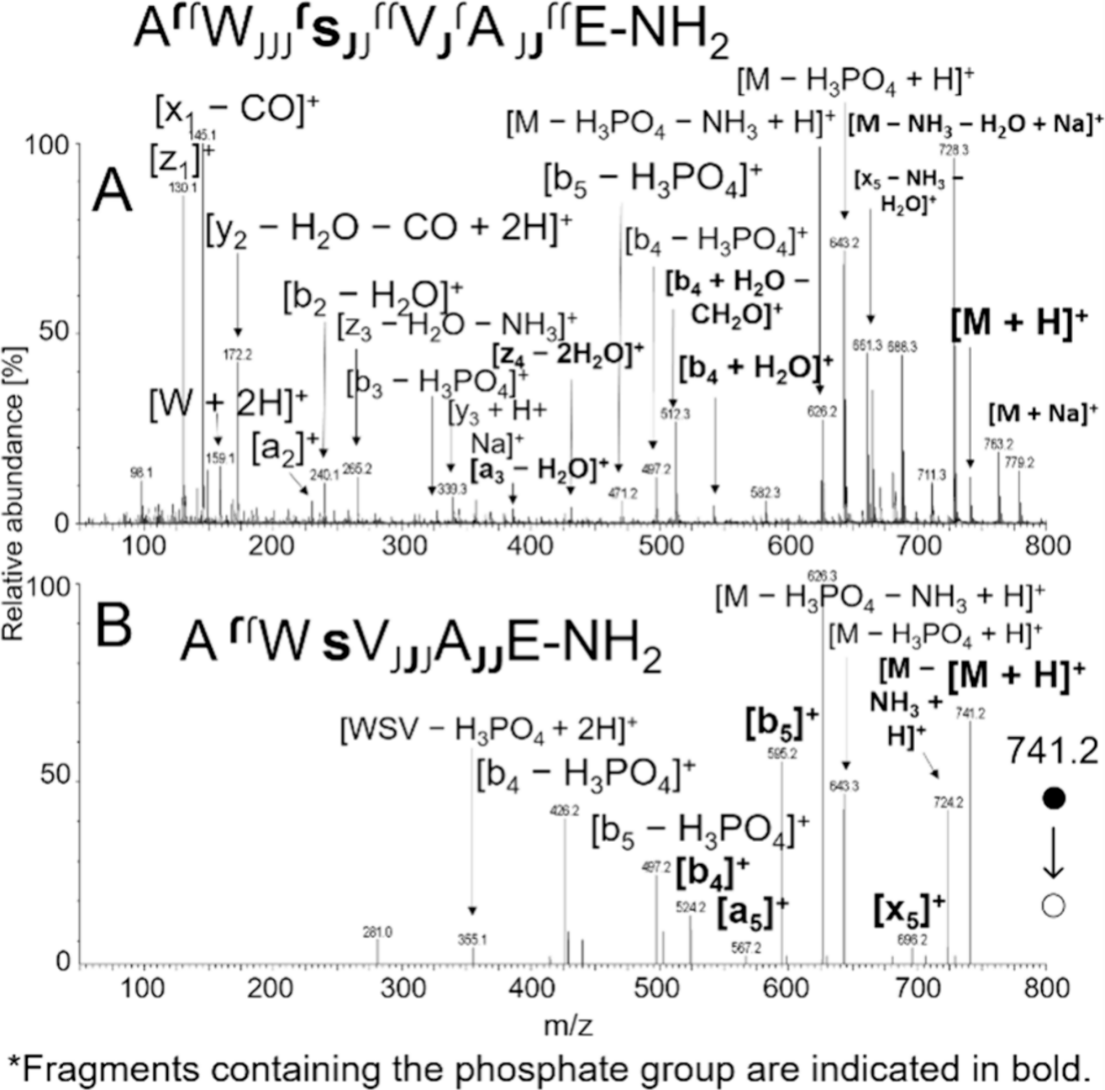
Full-scan soft X-ray APPI spectrum of phosphorylated AWsVAE-NH_2_ (A) and MS/MS CID (26 V) spectrum for [M + H]^+^ (B). The Waters ZSpray was operated in positive ion mode with the probe temperature set to 650 °C. The peptide was dissolved in Milli-Q water and infused from a syringe (10 μL/min) into a 40 μL/min Milli-Q water flow.

The radical-driven fragmentation pathways originate from radical precursors formed during the APPI process. However, these radical precursors were not detected in the spectra, likely due to their rapid decay into fragment ions. Peptide radical cations are generally less stable than their anionic counterparts.^53^ N–Cα peptide fragmentation has been previously studied in dopant-assisted VUV APPI.^35,54–56^ The formation of radical fragment ions was found to correlate with the amount of dopant introduced and the depletion of doubly protonated peptide ions.^55,56^ Photoionization of the dopant or other molecules with sufficiently low ionization energies leads to electron release, which can also occur from ion source surfaces via the photoelectric effect. These electrons participate in various reactions, contributing to the formation of radical anions, such as superoxide anion O_2_^•–^. The presence of O_2_^•–^ was confirmed in soft X-ray APPI operating in negative ion mode (Figure S2). Electrons or radical anions have been proposed to generate transient radical cations through electron capture or transfer to [M + 2H]^2+^ (Reactions 1 and 2), thereby facilitating N–Cα peptide fragmentation.^41^

1 $${\left[M+2H\right]}^{2+}+e^{-}\to {\left[M+2H\right]}^{•+}\to frag$$

2 $${\left[M+2H\right]}^{2+}+{O_{2}}^{•-}\to {\left[M+2H\right]}^{•+}+O_{2}\to frag$$

Another potential pathway for generating radical cation precursors involves electron detachment (Reaction 3), initiated by electrons with energies exceeding 10 eV.^57^

3 $${\left[M+nH\right]}^{n+}+e^{-}\to {\left[M+nH\right]}^{\left(n+1\right)•+}+2e^{-}\to frag$$

A hydrogen capture process (Reaction 4) has been proposed to explain N–Cα bond cleavages in peptides that predominantly form singly charged species in APPI.^56^ Similar fragmentation mechanisms are well documented in in-source decay (ISD) during matrix-assisted laser desorption/ionization (MALDI).^58,59^

4 $${\left[M+nH\right]}^{n+}+H^{•}\to {\left[M+\left(n+1\right)H\right]}^{n+•}\to frag$$

The ionization pathways leading to radical peptide fragments in soft X-ray APPI were not further investigated in this study. However, the detection of doubly protonated peptide ions (Figure 1) and superoxide anion (Figure S2) suggests that electron transfer, as described in Reaction 2, may contribute to peptide radical formation.

All proposed peptide fragmentation pathways originate from even-electron peptide cations, leading to their depletion, particularly in soft X-ray APPI. Furthermore, their reduced detection during photoionization (Figure 1) may be attributed to ion pair formation with excessive negatively charged species, resulting in the generation of undetectable neutral molecules.

### APPI Spectra of Peptides in Negative Ion Mode

Negative ion mode spectra of peptide standards (Section 2.2) were acquired using soft X-ray APPI and VUV APPI. In the absence of a photon source, the ZSpray ion source produced only nonabundant peptide ions (Figure 3A), suggesting that significant thermospray-like processes were not present. Deprotonated peptides dominated in full-scan mass spectra from both APPI sources. The fragmentation patterns were similar for both ionization methods, with major fragments corresponding to b-type ions, often accompanied by water or carbon monoxide losses (Figures 3B–C).

**Figure 3:**
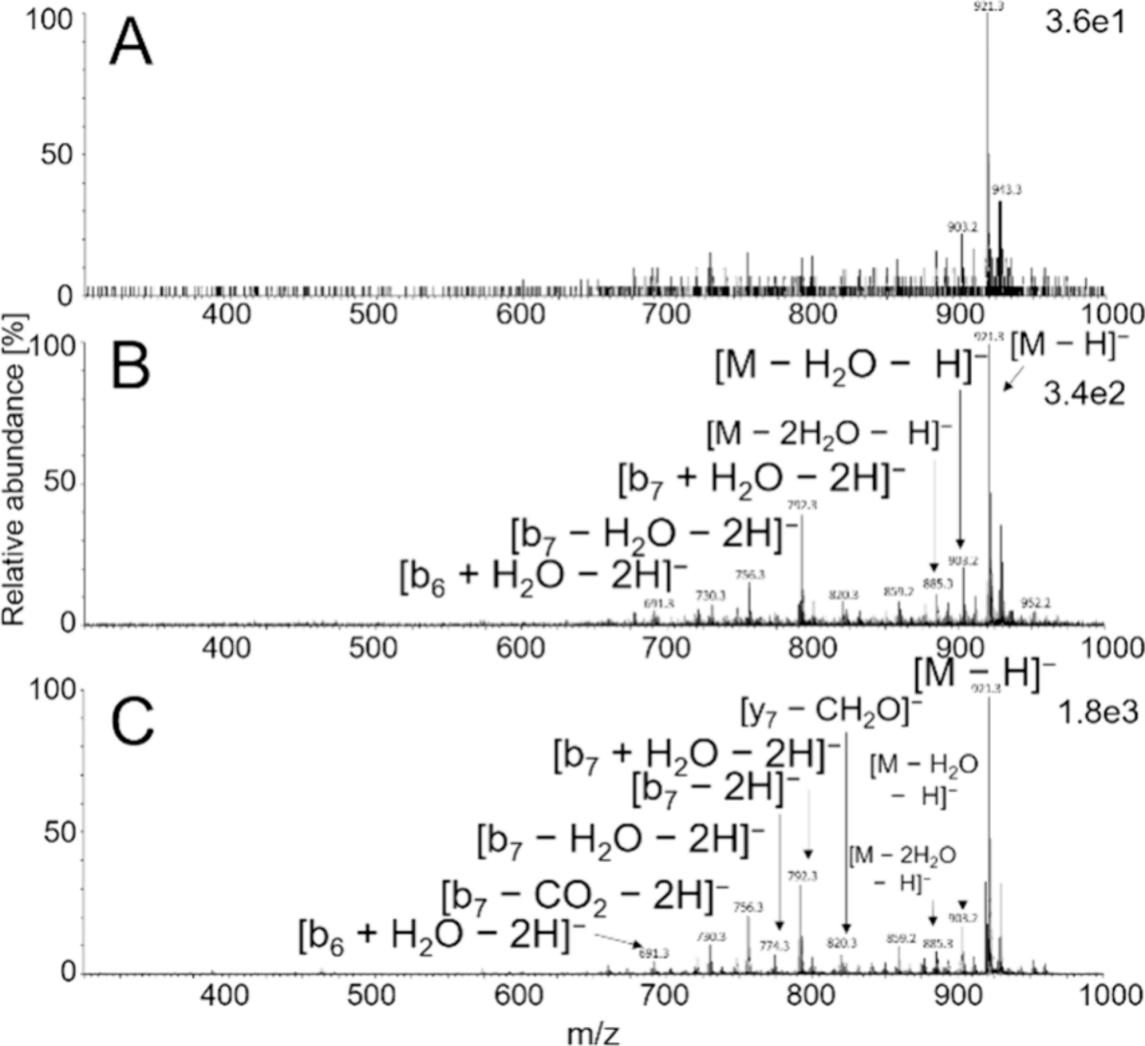
Full-scan mass spectra of AEFVEVTE obtained in soft X-ray APPI with the photon source turned off (A), VUV APPI (B), and soft X-ray APPI with the photon source turned on (C). The Waters ZSpray was operated in negative ion mode with the probe temperature set to 650 °C. The peptide was dissolved in Milli-Q water and infused from a syringe (10 μL/min) into a 40 μL/min Milli-Q water flow.

Regarding detection sensitivity, soft X-ray APPI consistently generated more abundant deprotonated molecules than VUV APPI for almost all peptide standards. The relative abundance of [M – H]^−^ ions in soft X-ray APPI compared to VUV APPI varied significantly, ranging from 1 to 85, depending on the peptide (Figure S3). The superior performance of soft X-ray APPI can be attributed to complex ionization processes in the ion source, particularly proton transfer and electron capture. Proton transfer occurs when the reaction Gibbs free energy Δ_r_G_acid_ of the analyte is lower (i.e., its acidity is higher) than that of the reagent. In negative ion mode, oxygen plays a critical role.^60^ With a positive electron affinity (EA 0.48 eV), oxygen can capture electrons released from dopants, solvents, or the metallic surface of the ion source,^60^ forming O_2_^•–^. The superoxide anion was detected in soft X-ray APPI (Figure S2) but not VUV APPI. The higher energy of soft X-ray photons likely generated more electrons, leading to an increased O_2_^•–^ formation. Due to its strong gas-phase basicity (Δ_r_G° 1450 kJ/mol),^61^ O_2_^•–^ readily accepts protons from solvents or analytes, facilitating the formation of their deprotonated species. This mechanism may explain, or at least contribute to, the higher abundance of deprotonated peptide molecules ([M – H]^−^) observed in soft X-ray APPI.

Comparison of the isotopic clusters of deprotonated molecules with theoretical models confirmed the formation of peptide radical anions (M^•–^) under both soft X-ray and VUV APPI conditions. This was validated by analyzing the fragmentation spectra of the first and second isotopic peaks of [M – H]^−^. While CID of the first isotopic peak ([M – H]^−^) yielded even-electron fragments, fragmentation of [M – H + 1]^−^, which included contributions from M^•–^, also generated minor signals corresponding to radical species (Figure S4). The relative abundance of the M^•–^ signals varied depending on the peptide structure, typically ranging between 5% and 10% of the intensity of the deprotonated molecule. A greater formation of radical anions was observed with soft X-ray APPI compared to VUV APPI (Figure S5). This enhanced generation of peptide radical anions in soft X-ray APPI may contribute to the overall higher intensity of [M – H]^−^. The formation of deprotonated peptides may be further explained by resonant electron capture by neutral peptides, leading to hydrogen dissociative loss,^62–65^ as described in Reaction 5.

5 $$M+e^{-}\to M^{•-}\to {\left[M-H\right]}^{-}+H^{•}$$

Peptides containing the aromatic amino acids tryptophan and phenylalanine generated low-abundance oxidized products [M – H + O]^−^ (Figure S6), likely formed through substitution reaction involving either M^•–^ with O_2_ or M with O_2_^•–^ (Reactions 6 and 7).^60^

6 $$M+{O_{2}}^{•-}\to {\left[M+O-H\right]}^{-}+HO^{•}$$

7 $$M^{•-}+O_{2}\to {\left[M+O-H\right]}^{-}+HO^{•}$$

A detailed spectral analysis also revealed the formation of hydrogen-deficient anions [M – 3H]^−^ and [M – 2H]^•–^ (Figure 4).

**Figure 4:**
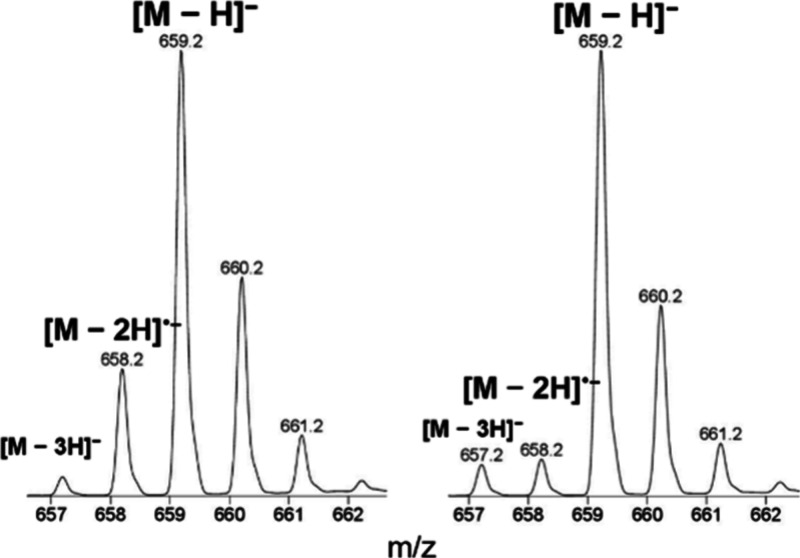
Sections of soft X-ray (left) and VUV APPI (right) full-scan mass spectra of AWSVAE-NH_2_ showing [M – H]^−^, [M – 2H]^•–^ and [M – 3H]^−^ ions. The peptide was dissolved in Milli-Q water and infused from a syringe (10 μL/min) into a 40 μL/min Milli-Q water flow. The probe temperature was set to 650 °C.

The formation of [M – 3H]^−^ has previously been reported for amino acids in reactive plasma^66^ and attributed to oxidative reactions involving hydroxyl radical HO^•^ ^67^ (Reactions 8 and 9, Scheme S1).

8 $${\left[M-H\right]}^{-}+HO^{•}\to {\left[M-2H\right]}^{•-}+H_{2}O$$

9 $${\left[M-2H\right]}^{•-}\to {\left[M-3H\right]}^{-}+H^{•}$$

The radical anion [M – 2H]^•–^ was detected in both VUV and soft X-ray APPI (Figure 4), with significantly higher formation observed in soft X-ray APPI. Compared to VUV APPI, the abundance of [M – 2H]^•–^ in soft X-ray APPI was higher by a factor of 1 to 520 (Figure S3). To our knowledge, this is the first study to report the detection of such species in an ion source. Several pathways may explain the formation of [M – 2H]^•–^. One possibility is the loss of a hydrogen molecule from M^•–^, as described in Reaction 10. This type of reaction has previously been reported for amino acids following electron capture.^68,69^

10 $$M^{•-}\to {\left[M-2H\right]}^{•-}+H_{2}$$

The [M – 2H]^•–^ ions may also form through hydrogen abstraction from [M – H]^−^ by gas-phase radicals, such as H^•^,^70,71^ or HO^•^ ^72^ (Reaction 11).

11 $${\left[M-H\right]}^{-}+H^{•}/OH^{•}\to {\left[M-2H\right]}^{•-}+H_{2}/H_{2}O$$

Another potential precursor for [M – 2H]^•–^ is the doubly deprotonated molecule, [M – 2H]^2–^. The loss of an electron from these doubly charged, even-electron species (Reactions 12–14) has been reported during ion activation by various techniques, including electron detachment dissociation (EDD),^26^ negative electron-transfer dissociation (NETD),^73,74^ UVPD,^24,73,75,76^ and electron photodetachment dissociation (EPD).^77,78^

12 $${\left[M-2H\right]}^{2-}+e^{-}\to {\left[M-2H\right]}^{•-}+2e^{-}$$

13 $${\left[M-2H\right]}^{2-}+A^{•+}\to {\left[M-2H\right]}^{•-}+A$$

14 $${\left[M-2H\right]}^{2-}+hv\to {\left[M-2H\right]}^{•-}+e^{-}$$

Doubly deprotonated peptides are typically not observed in APPI; however, a closer inspection of both VUV and soft X-ray APPI spectra revealed their presence, albeit at noise level. It suggests that the formation of radial anions from [M – 2H]^2–^ might still be possible. For AEFVEVTK, the generation of [M – 2H]^•–^ from [M – 2H]^2–^ through interaction with 213 nm photons was confirmed experimentally (Figure S7). The formation efficiency of [M – 2H]^•–^ was peptide-dependent, with the highest efficiency observed in tryptophan-containing peptides. In these cases, [M – 2H]^•–^ reached 20–35% of the [M – H]^−^ abundance, whereas in other peptides, it remained below 15% (Figure S8). The increased yield of radical anions in tryptophan-containing peptides may be attributed to radical stabilization within the aromatic system of tryptophan.^75–77,79^ Additionally, the relatively high electron affinity and low ionization energy of tryptophan make these peptides more susceptible to electron attachment or detachment.^80^

The formation efficiency of [M – 2H]^•–^ was also influenced by probe temperature and sample solution flow rate. The highest yield of [M – 2H]^•–^ was observed at a relatively low flow rate of 40 μL/min, as discussed in more detail in Text S2. The abundance of [M – 2H]^•–^ increased exponentially with rising temperature, reaching its maximum at the highest tested temperature of 650 °C (Figure S9 and S10). This indicates that the temperature plays a crucial role in the formation of [M – 2H]^•–^. At elevated temperatures, radical-driven reactions may become more prevalent, leading to fragmentation and dehydrogenation. It is plausible that hydrogen elimination from the deprotonated molecule occurs at higher temperatures, as described by Reaction 15; however, experimental evidence for this process remains lacking.

15 $${\left[M-H\right]}^{-}\overset{T}{\to }{\left[M-2H\right]}^{•-}+H^{•}$$

Despite the significant difference in photon energies between VUV and soft X-ray APPI, full-scan mass spectra in negative mode showed no major differences in peptide fragmentation patterns. The formation of b-fragments increased with higher ion source probe temperatures, suggesting that thermal mechanisms, rather than photon energy, are primarily responsible for their generation (Figure S11). In contrast, the formation of c-, z-type, and a-, x-type fragments remained unaffected by temperature. The c-, z-fragments are well-known products of [M – H]^−^ dissociation,^14,17,22^ and their formation is attributed to resonant electron capture (>5 eV) by a neutral peptide, leading to the generation of a metastable radical anion that undergoes immediate fragmentation.^62,64,65^ Since a-, x- fragments are produced through the CID of peptide radical anions, their formation within the ion source can be linked to collisional activation. The yields of c-, z-, and a-, x- fragments were comparable in both VUV and soft X-ray APPI (Figure S11), indicating that the lower energy VUV photons are sufficient to overcome the energetic barriers required for peptide cleavage. Soft X-ray photons induced slightly higher peptide fragmentation rates, but only for longer peptides such as QTALVELL-E and -F.

### CID of [M – 2H]^•–^

In proteomics, fragmenting odd-electron precursors often yields unique cleavage patterns that provide detailed structural insights, aiding the identification of peptide sequences and their modifications. Soft X-ray APPI facilitates the formation of radical anions [M – 2H]^•–^, which can be used for peptide structural analysis. To explore this potential, we examined the fragmentation spectra of these ions and compared them to those of [M – H]^−^.

The CID spectra of singly charged deprotonated peptides [M – H]^−^ have been extensively studied.^13–21^ Fragmentation typically occurs through peptide backbone cleavages at C–N and N–Cα bonds, alongside neutral losses and side-chain cleavages. The elimination of small neutral molecules is more pronounced in shorter peptides.^12,21^ For example, the shortest peptide analyzed in this work, SLGF, exhibited combined losses of water, formaldehyde from serine, carbon dioxide from the C-terminus, a 2-methylpropyl radical from leucine, and a benzyl radical from phenylalanine (Figure 5). Longer peptides generally showed fewer neutral losses and favored peptide backbone fragmentation. However, some neutral losses remained relatively common even in longer peptides. The most prominent peptide backbone cleavages generated abundant c-, z-, and y-ions. Notably, the formation of b_n–1_ or b_n–1_ + H_2_O fragments was highly specific to peptides with glutamic acid at their C-termini.

**Figure 5:**
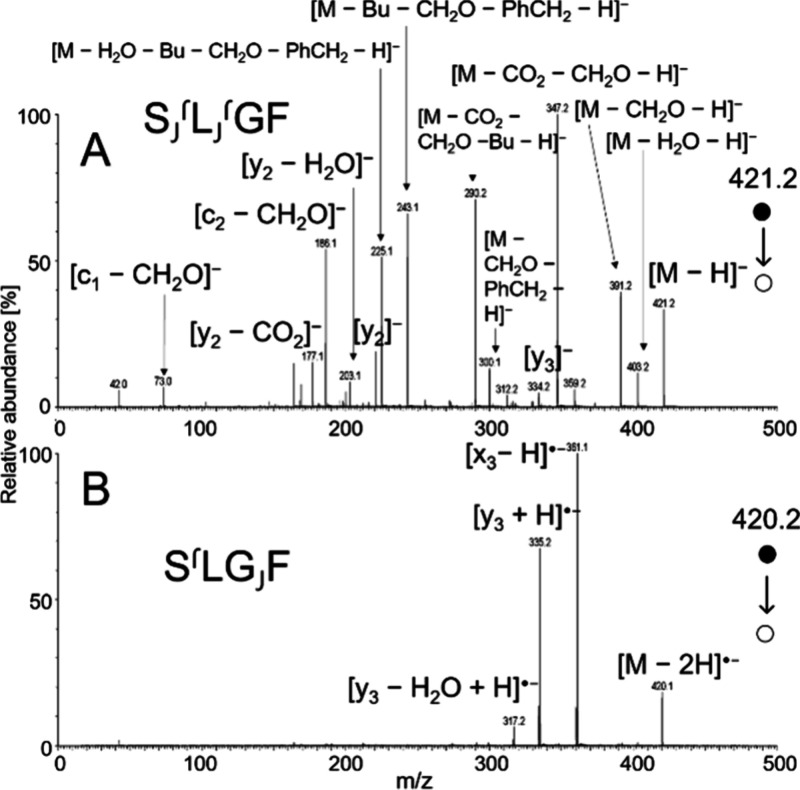
CID spectra of SLGF obtained using VUV APPI at 23 V for [M – H]^−^ (A) and soft X-ray APPI at 18 V for [M – 2H]^•–^ (B).

The fragmentation spectra of [M – 2H]^•–^ differed significantly from those of deprotonated molecules, with notable differences observed for the short peptide SLGF (Figure 5). While the deprotonated molecule primarily produced fragments corresponding to neutral losses, collisional activation of [M – 2H]^•–^ triggered entirely different fragmentation channels, resulting in the abundant formation of C-terminal fragments y_3_ and x_3_.

Radical-driven fragmentation pathways played a particularly significant role in the fragmentation of peptides containing tryptophan. Computational studies revealed that the radical is localized on the indole nitrogen,^26,75,76^ where it is presumably mobile,^81^ facilitating radical-driven fragmentation pathways. In AWSVAE-NH_2_, collisional activation of [M – 2H]^•–^ produced abundant a_5_-, x_2,3, 4,5_-, z_1,2,3,4_-_,_ and y_5_-products. In contrast, fragmentation of [M – H]^−^ precursor generated an entirely different spectrum, dominated by neutral loss of formaldehyde and exhibiting minimal backbone cleavages (Figure 6). The high abundance of a- and x-series ions in the [M – 2H]^•–^ spectrum can be attributed to the preferential cleavage of the Cα-C bond, alongside the suppression of N–Cα and C–N bond cleavages, as previously demonstrated for N-radical-centered peptides.^82^ The a- and x-ion series were detected as both deprotonated molecules and radical anions. Consistent with previous studies, the detection of a·- and x-series was slightly more frequent than that of the a- and x·-series.^26,78,82^

**Figure 6:**
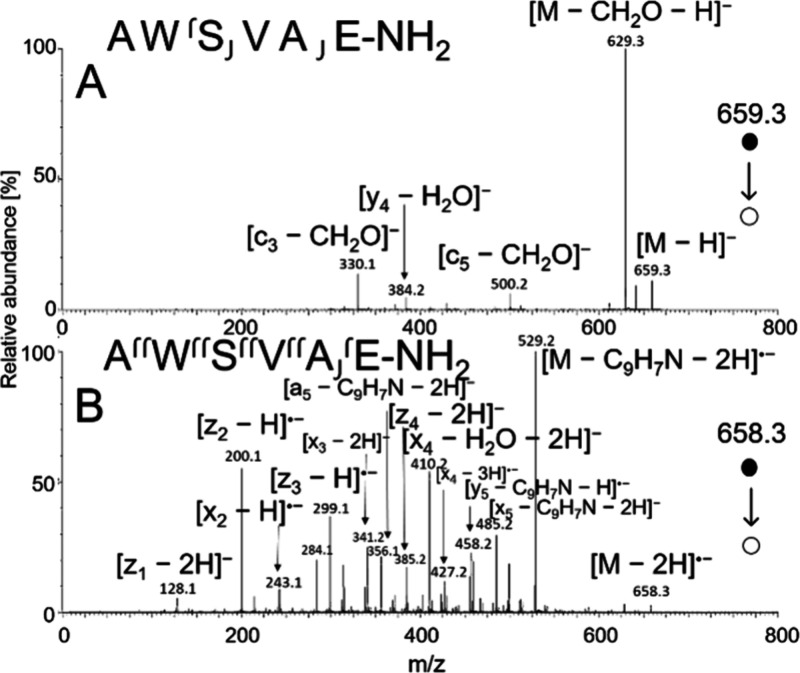
CID spectra of AWSVAE-NH_2_ obtained using VUV APPI at 34 V for [M – H]^−^ (A) and soft X-ray APPI at 28 V for [M – 2H]^•–^ (B).

As demonstrated in numerous studies, radical-driven fragmentation pathways serve as a powerful tool for localizing PTMs.^78^ Here, we investigated the utility of [M – 2H]^•–^ for pinpointing phosphorylation on serine in AWsVAE-NH_2_. Figure 7 compares the CID spectra of AWsVAE-NH_2_ for the [M – H]^−^ and [M – 2H]^•–^ precursors. While the deprotonated molecule primarily exhibited a neutral loss of phosphoric acid, the [M – 2H]^•–^ fragmentation spectrum contained numerous informative fragments for phosphorylation site localization. These included a_3,5_-, b_5_-, c_4,5_-, y_5_-, and z_5_-fragments, all of which retained the phosphate group.

**Figure 7:**
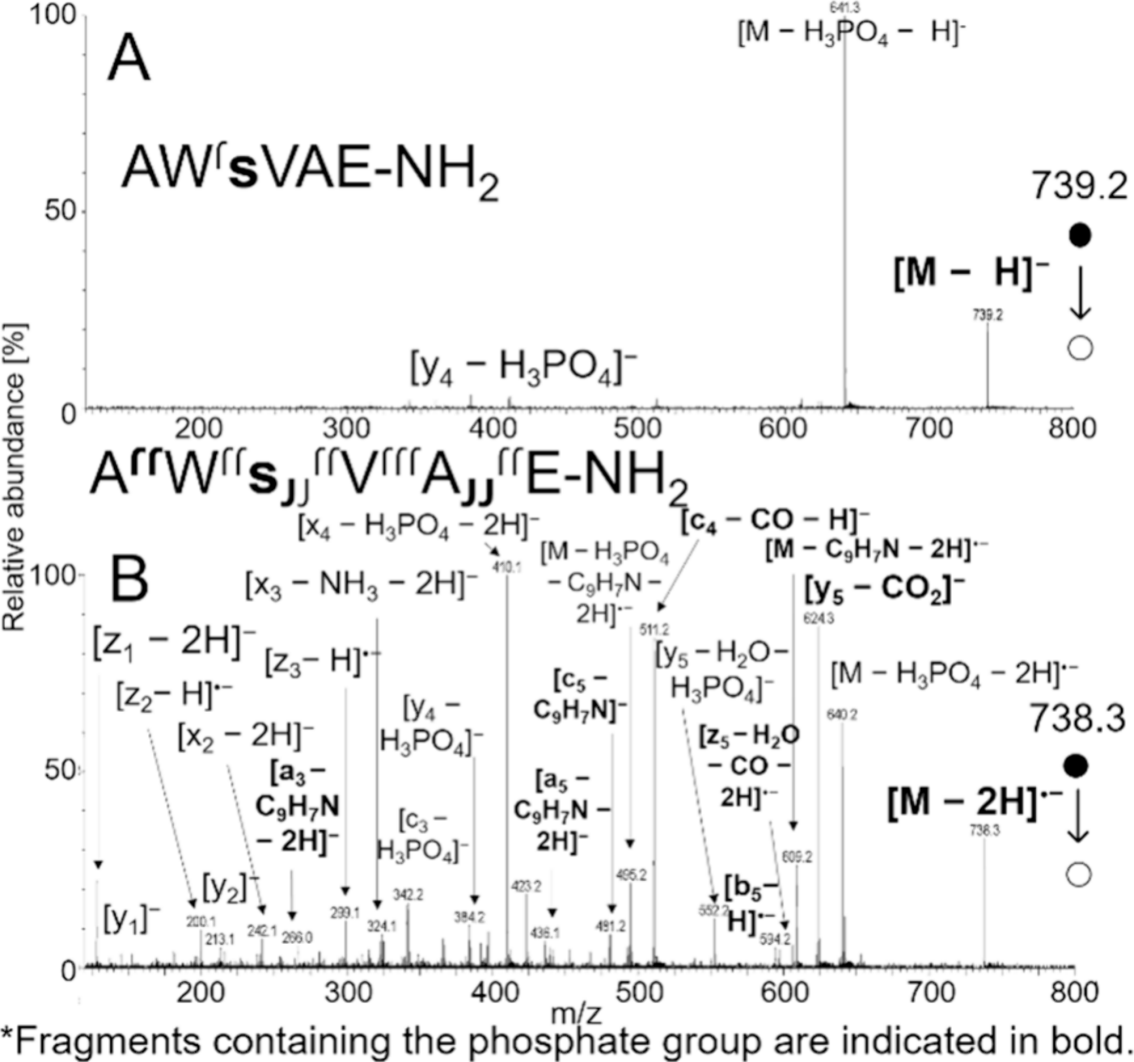
CID spectra of phosphorylated AWsVAE-NH_2_ obtained using VUV APPI at 38 V for [M – H]^−^ (A) and soft X-ray APPI at 28 V for [M – 2H]^•–^ (B).

Lipidation is a critical peptide modification that enhances membrane anchoring and plays key roles in various cellular functions. However, lipidated peptides often exhibit poor fragmentation in CID, making it challenging to generate informative fragment ions for precise modification site identification. To address this, we compared the CID spectra of GVSGSK-NH_2_ peptides myristoylated of palmitoylated at their N termini for both [M – H]^−^ and [M – 2H]^•–^ precursors. The spectra for Myr-GVSGSK-NH_2_ are shown in Figure 8. Both precursors readily eliminated formaldehyde from the serine residue. However, beyond neutral loss fragments, the deprotonated molecule produced only a single backbone cleavage product, c_4_ – CH_2_O. In contrast, the radical anion [M – 2H]^•–^ fragmented extensively, generating a rich spectrum of fragments, particularly a- and x-ions associated with radical-driven fragmentation pathways. The N-terminal fragments (a- and c-series) retained the myristoylation. A similar fragmentation pattern was observed for Palm-GVSGSK-NH_2_ (Figure S12). These findings demonstrate that the fragmentation of [M – 2H]^•–^ is advantageous for preserving PTMs, enabling more accurate localization and characterization of lipidation sites in mass spectrometry analysis.

**Figure 8:**
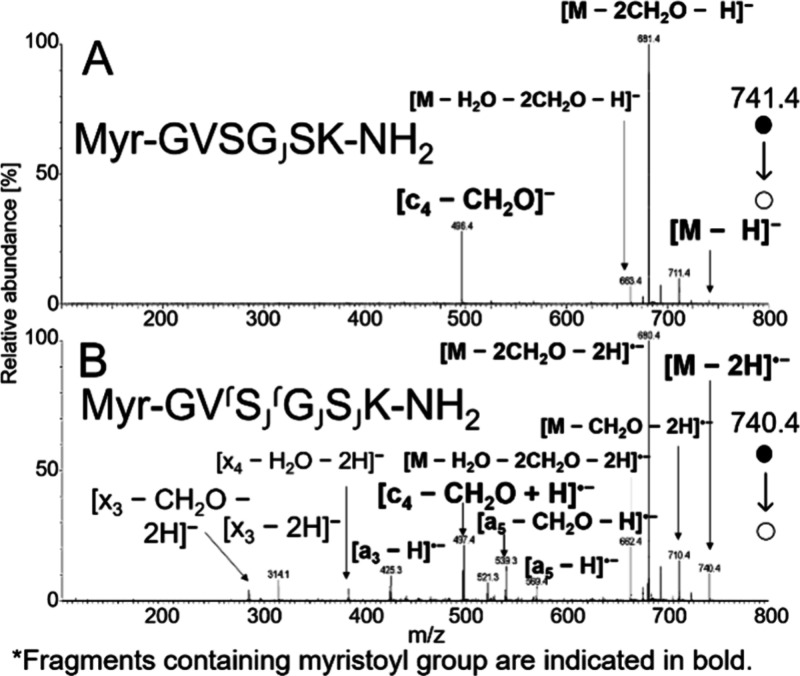
CID spectra of *N*-myristoyl GVSGSK-NH_2_ obtained using VUV APPI at 34 V for [M – H]^−^ (A) and soft X-ray APPI at 28 V for [M – 2H]^•–^ (B).

## Conclusions

The performance of soft X-ray APPI was evaluated on 21 peptides in both positive and negative ion modes, with a direct comparison to conventional VUV APPI. In positive ion mode, high-energy soft X-ray photons facilitated the depletion of [M + H]^+^ and promoted the cleavage of Cα-C and N–Cα bonds, generating structurally informative fragments. In negative ion mode, [M – H]^−^ was the dominant species in most peptide mass spectra with significantly enhanced detection using soft X-ray APPI. This enhancement was likely attributed to the increased presence of O_2_^•–^, which effectively facilitated peptide deprotonation.

To our knowledge, soft X-ray APPI is the first ion source capable of generating peptide hydrogen-deficient radical anions ([M – 2H]^•–^), highlighting its unique performance in negative ion mode. While the formation of peptide [M – 2H]^•–^ has previously been observed in MS/MS experiments using electron or photon activation methods, or under CID typically requiring peptide modification, soft X-ray APPI enables their formation under atmospheric pressure, eliminating the need for advanced MS^n^ techniques or chemical modifications. The ionization processes induced by soft X-ray photons are complex, and likely involve interactions with electrons and reactive radical species. Although several potential pathways for [M – 2H]^•–^ formation have been proposed, further studies are necessary to confirm these mechanisms and clarify their contributions.

The generation of [M – 2H]^•–^ was influenced by flow rate, probe temperature, and peptide sequence, with the highest production observed at a relatively low flow rate (up to 50 μL/min) and the maximum tested probe temperature (650 °C). Peptide containing tryptophan exhibited the highest yields of [M – 2H]^•–^ among all analyzed sequences.

The generation of peptide radical ions has long been of particular interest, due to their ability to provide detailed structural insights, particularly in the localization of PTMs. The [M – 2H]^•–^ ions produced by soft X-ray APPI were stable enough for isolation and CID analysis. The CID spectra of [M – 2H]^•–^ showed preferential cleavage of Cα-C bonds, with suppressed neutral losses from side chains. This fragmentation pattern is particularly advantageous for PTM localization, as demonstrated for AWsVAR-NH_2_ phosphorylated on serine and GVSGSK-NH_2_ myristoylated or palmitoylated at its N-terminus.

This study highlights soft X-ray APPI as a powerful tool for advanced peptide structural analysis, particularly in PTMs characterization, while also enhancing our understanding of ionization and peptide fragmentation in the often-overlooked yet promising negative ion mode. The unique capability of soft X-ray APPI to generate stable radical ions suggests that its potential applications could extend beyond proteomics, offering the potential for the analysis of other biomolecules, such as nucleic acids and short oligonucleotides. Thus, soft X-ray APPI could complement existing ionization techniques in broader biomolecular research.

## Supplementary Material



## Notes

Additional texts with experimental details, including peptide synthesis, flow rate optimization, calculations of peptide ion representation, full-scan and fragmentation mass spectra, peptide detection sensitivity, and the effect of probe temperature, flow rate, or peptide sequences on the detected peptide ions (PDF)
