# Supplementary material for: Peptide Analysis by Soft X‑ray Atmospheric Pressure Photoionization Mass Spectrometry
Source: J Am Soc Mass Spectrom. 2025 May 19;36(6):1286–95. doi: 10.1021/jasms.5c00037 (PMC12142675; doi:10.1021/jasms.5c00037)
Supplement: Supplementary file 1 [file js5c00037_si_001.pdf]

Supporting Information

Peptide Analysis by Soft X-ray Atmospheric Pressure Photoionization Mass Spectrometry

Simona Sedláčková [1,2], Juha-Pekka Hieta [3], Miroslava Blechová [1] and Josef Cvačka [1,2*]

[1] Institute of Organic Chemistry and Biochemistry of the Czech Academy of Sciences, Flemingovo nám. 2, CZ-166 10 Prague 6, Czech Republic

[2] Department of Analytical Chemistry, Faculty of Science, Charles University in Prague, Hlavova 2030/8, CZ-128 43 Prague 2, Czech Republic

[3] Drug Research Program and Division of Pharmaceutical Chemistry and Technology, Faculty of Pharmacy, University of Helsinki, P.O. Box 56, FI-00014 Helsinki, Finland

* Email: josef.cvacka@uochb.cas.cz

Text S1: Peptide synthesis 2

Text S2: Effect of flowrate on the peptide ions creation 2

Figure S1. Effect of flowrate on the peptide ions creation 3

Text S3: Calculation of the peptide ions representation 3

Figure S2. Background mass spectrum in soft X-ray APPI. 4

Figure S3. Peptide detection sensitivity 5

Figure S4. Peptide $[M-H]^-$ and $M^{\bullet-}$ CID spectra 6

Figure S5. The relative representation of peptides $M^{\bullet-}$ in soft X-ray and VUV APPI. 6

Figure S6. Mass spectra of SLGF in VUV and soft X-ray APPI. 7

Scheme 1: Possible hydroxyl radical reaction in soft X-ray APPI 7

Figure S7. Peptide $[M-2H]^{2-}$ UVPD spectra. 8

Figure S8. Relative representation of peptides $[M-2H]^{\bullet-}$ in soft X-ray APPI. 9

Figure S9. Effect of probe temperature on the intensity ratio of $[M-2H]^{\bullet-}$ to $[M-H]^-$ 9

Figure S10. Effect of probe temperature on the creation of peptide $[M-2H]^{\bullet-}$ 10

Figure S11. Effect of the probe temperature on the creation of different peptide ions 10

Figure S12. Peptide $[M-H]^-$ and $[M-2H]^{\bullet-}$ CID spectra 11

SI References 11

Text S1: Peptide synthesis

Peptides were synthesized using a Liberty Blue solid-phase synthesizer (CEM Corporation, Charlotte, NC, USA) via stepwise coupling of Fmoc-protected amino acids to the growing chain on the following resins: Fmoc-Arg(Pbf)-WANG (100–200 mesh, 0.64 mmol/g), Fmoc-Lys (Boc)-WANG (100–200 mesh, 0.65 mmol/g), FmocGlu(OtBu)-WANG (200–400 mesh, 0.62 mmol/g), and Fmoc-Phe-WANG (200–400 mesh, 0.67 mmol/g). Fully protected peptides were synthesized following a protocol that involved sequential removal of the Nα-Fmoc protecting group using 20% piperidine in DMF and coupling mediated by DIC/Oxyma in DMF. Upon completion of synthesis, deprotection and cleavage of the linear peptides from the resins were performed simultaneously using a cleavage mixture of TFA/H_2O/TIS (95:2.5:2.5) . After cleavage, each resin was washed with dichloromethane, and the combined TFA filtrates were evaporated under nitrogen at room temperature. The precipitated residues were triturated with *tert*-butyl methyl ether, collected via suction, and dried via lyophilization. The peptides were purified on HPLC column ReproSil Gold 120 (C18, 5 µm, 250 x 20 mm) purchased from Dr. Maisch (Ammerbuch-Entringen, Germany) with a mobile phase consisting of acetonitrile and water (0.1% TFA). Separation was carried out on a Waters HPLC system equipped with a Delta 600 pump and a 2489 UV/VIS detector.

Text S2: Flowrate

The highest $[M - H]^-$ and $[M - 2H]^{\bullet -}$ signals were achieved at 40 µL/min, as shown in Figure S1. Increasing the flow rate beyond this point led to a decline in $[M - H]^-$ and $[M - 2H]^{\bullet -}$ formation, favoring the production of $[M + TFA - H]^-$. As the flow rate increased, the concentration of trifluoroacetic acid (TFA), a common impurity, also rose in the ion source, resulting in a stronger $[TFA - H]^-$ signal at higher flow rates. The deprotonation of TFA (ΔG_{acid} 1328 kJ/mol) can occur via reaction with $O_2^{\bullet -}$, thereby reducing the availability of $O_2^{\bullet -}$ for other ionization processes, such as peptide deprotonation. Additionally, reduced ionization efficiency at elevated flow rates is often attributed to incomplete desolvation of the solvent. This limitation can be partially mitigated by increasing the probe temperature (S3).

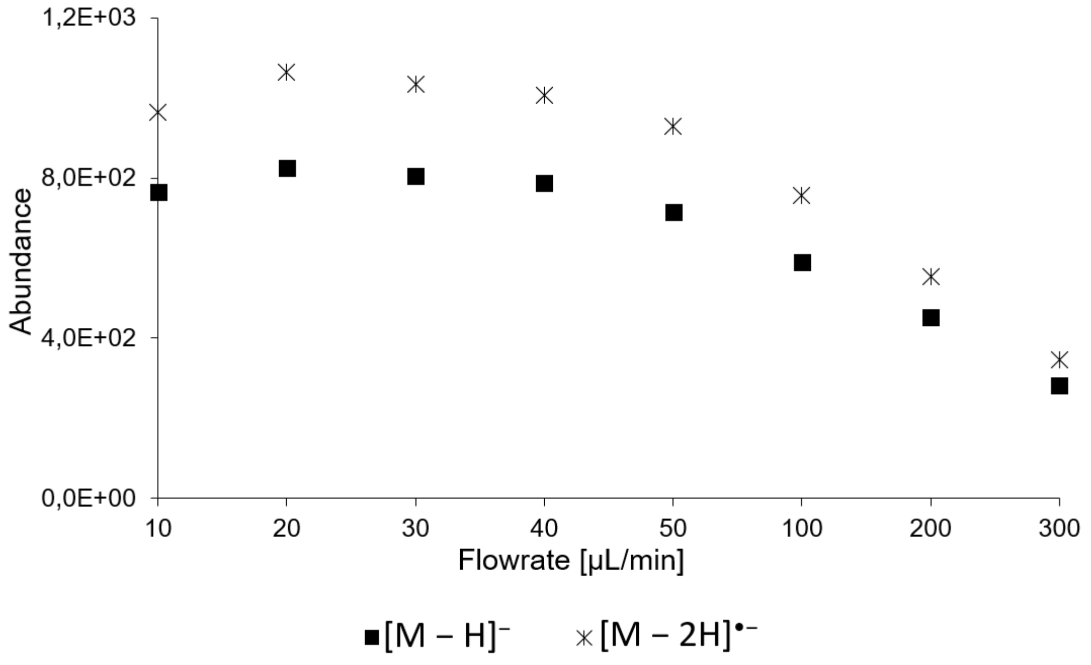

Figure S1. Effect of the solvent flowrate on the AWSVAR $[M-H]^-$ and $[M-2H]^{\bullet-}$ signals. The peptide AWSVAR dissolved in Milli-Q water was infused from a syringe (10 µL/min) into the Milli-Q water flow (10–300 µL/min). The probe temperature was set to 650 °C.

Text S3: Calculation of the peptide ions representation

The resolving power of the mass spectrometer used in this study was insufficient to fully resolve the isotopologues of $[M-H]^-$, $[M-2H]^{\bullet-}$, $[M-3H]^-$ and $M^{\bullet-}$. Consequently, the isotopic profiles overlapped. The $[M-2H]^{\bullet-}$ peak represented the combined signal of $[M-2H]^{\bullet-}$ and isotope A of $[M-3H]^-$. The $[M-H]^-$ peak comprised the sum of $[M-H]^-$, isotope A of $[M-2H]^{\bullet-}$, and isotope A + 1 of $[M-3H]^-$. Similarly, the $M^{\bullet-}$ peak encompassed the combined signals of $M^{\bullet-}$, isotope A of $[M-H]^-$, and isotope A + 1 of $[M-2H]^{\bullet-}$.

The true abundances of monoisotopic peaks representing $[M-H]^-$, $[M-2H]^{\bullet-}$, and $M^{\bullet-}$ were calculated as follows:

$$\mathrm{real}[\mathrm{M}-2\mathrm{H}]^{\bullet-} = \mathrm{m}[\mathrm{M}-2\mathrm{H}]^{\bullet-} - (\mathrm{ri}[\mathrm{M}-3\mathrm{H}]^{-}\ \mathrm{isotope\ A} \cdot \mathrm{m}[\mathrm{M}-3\mathrm{H}]^{-})$$

$$\mathrm{real}[\mathrm{M}-\mathrm{H}]^{-} = \mathrm{m}[\mathrm{M}-\mathrm{H}]^{-} - (\mathrm{ri}[\mathrm{M}-2\mathrm{H}]^{\bullet-}\ \mathrm{isotope\ A} \cdot \mathrm{real}[\mathrm{M}-2\mathrm{H}]^{\bullet-}) - (\mathrm{ri}[\mathrm{M}-3\mathrm{H}]^{-}\ \mathrm{isotope\ A}+1 \cdot \mathrm{m}[\mathrm{M}-3\mathrm{H}]^{-})$$

$$\mathrm{realM}^{\bullet-} = \mathrm{real}[\mathrm{M}-\mathrm{H}]^{-} - (\mathrm{ri}[\mathrm{M}-2\mathrm{H}]^{\bullet-}\ \mathrm{isotope\ A} \cdot \mathrm{real}[\mathrm{M}-2\mathrm{H}]^{\bullet-}) - (\mathrm{ri}[\mathrm{M}-3\mathrm{H}]^{-}\ \mathrm{isotope\ A}+1 \cdot \mathrm{m}[\mathrm{M}-3\mathrm{H}]^{-})$$

Where real = real abundance; m = measured abundance; ri = relative intensity of isotope to molecular ion obtained from simulated isotope pattern. The measured abundances were obtained from full scan mass spectra. The ri values for each peptide were determined through simulation using mMass software (S4). Since no peaks corresponding to $[M-4]^-$ were detected, the abundances of $[M-3H]^-$ were considered real, without contributions from other ion.

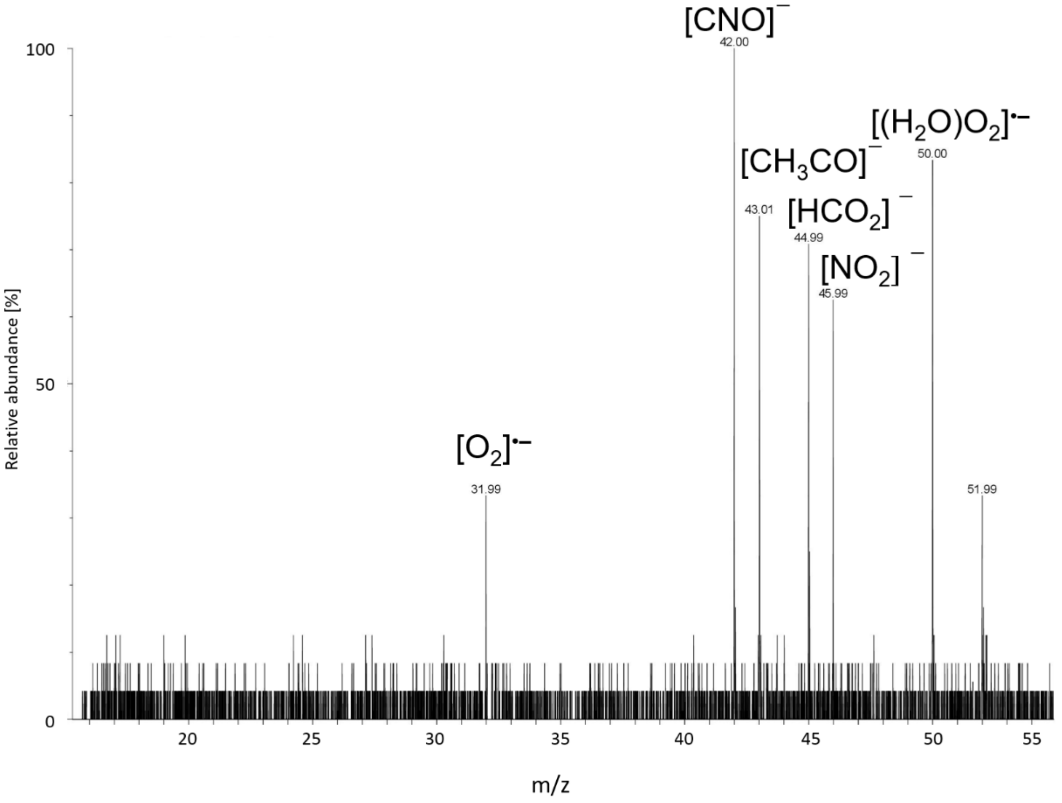

Figure S2. Soft X-ray APPI background spectrum of negative ions. Milli-Q water was introduced using a syringe at a flow rate of 50 μL/min

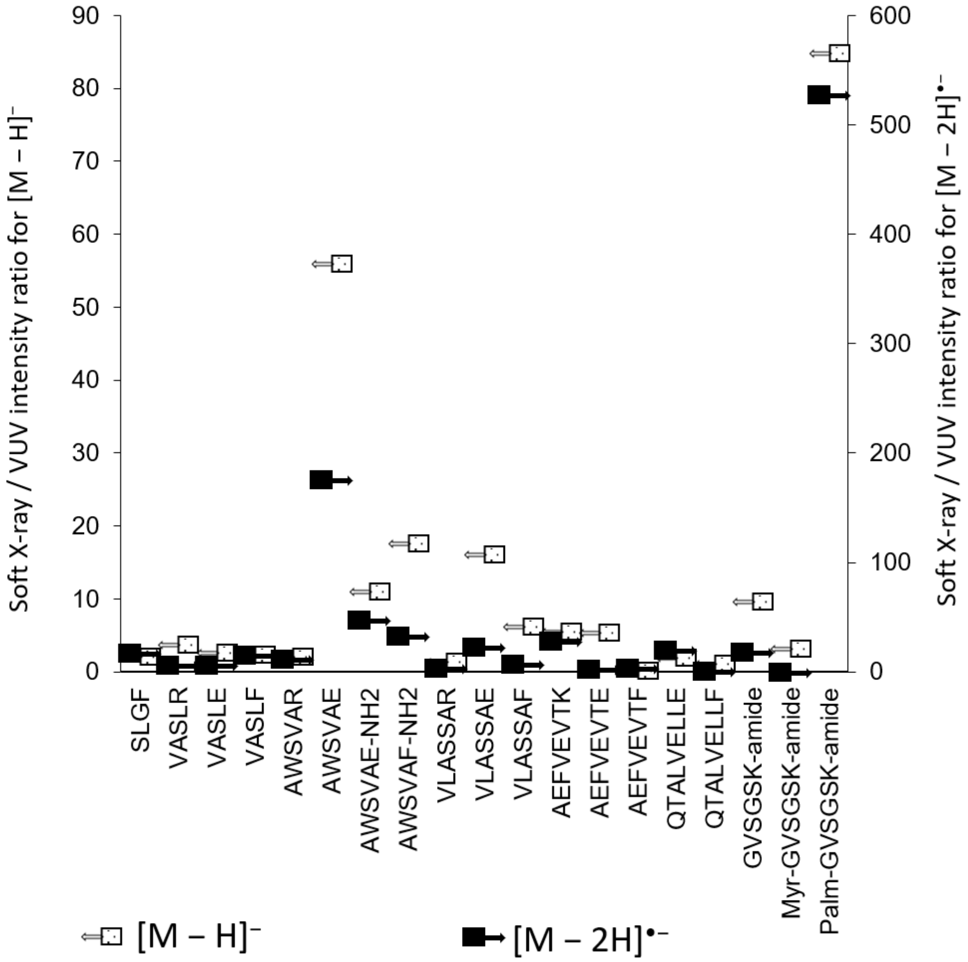

Figure S3. Peptide detection sensitivity presented as the intensity ratio of $[M - H]^{-}$ (or $[M - 2H]^{\bullet -}$) calculated from full scan mass spectra obtained by soft X-Ray APPI to intensity $[M - H]^{-}$ (or $[M - 2H]^{\bullet -}$) calculated from full scan mass spectra VUV APPI. The calculated intensities were achieved based on measured abundances and simulated peptide ion isotopic distributions.

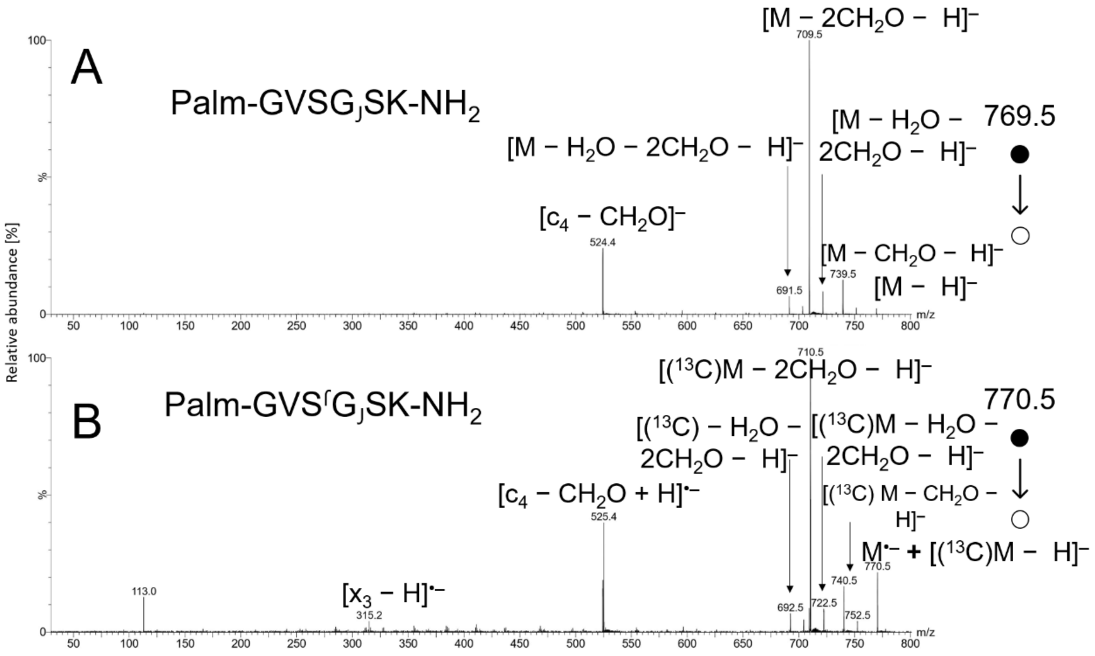

Figure S4. Soft X-ray APPI CID spectra of (A) [M − H]$^-$ (CID 34 V) and (B) M$^{•-}$ (CID 30 V) (corresponding to the mixture of [M − H]$^-$ isotope and M$^{•-}$) for Palm-GVSGSK-NH2.

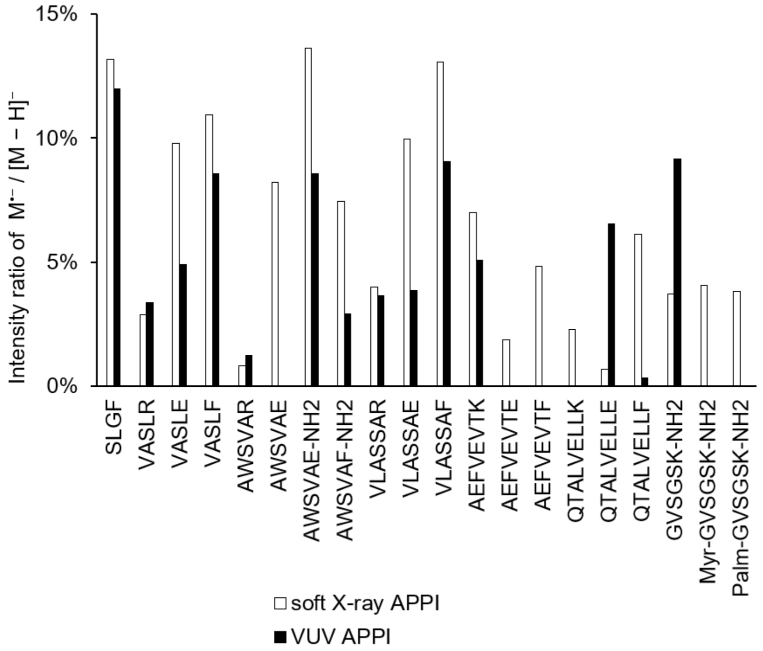

Figure S5. The intensity ratio of M$^{•-}$ to [M − H]$^-$ calculated from full scan soft X-ray and VUV APPI mass spectra. The ion abundances were corrected for the contribution of isobaric ions.

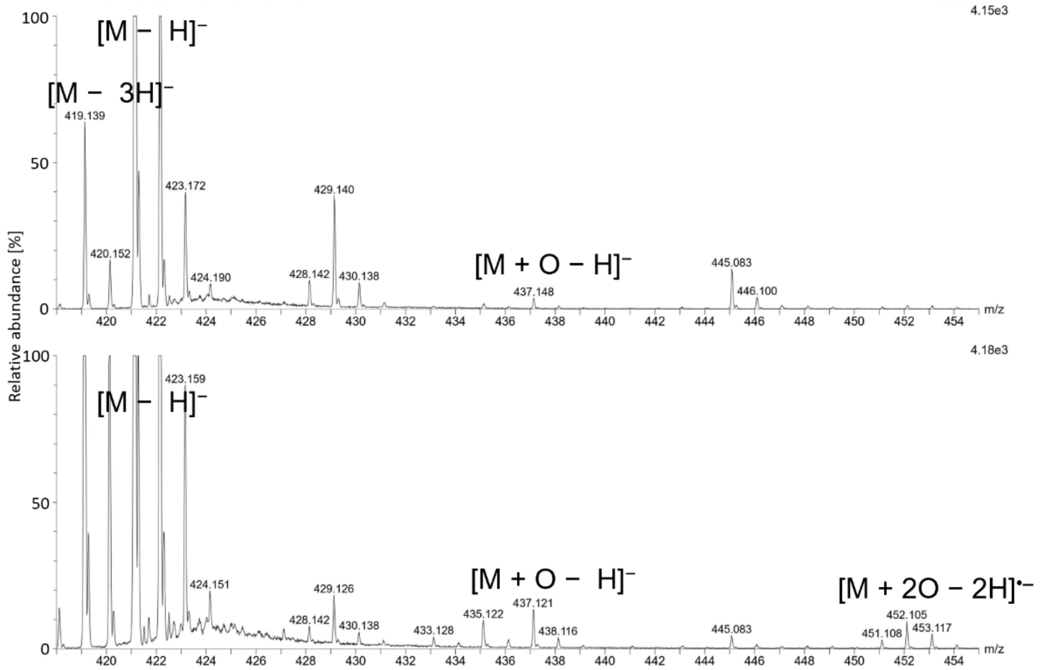

Figure S6. The full scan mass spectra of SLGF recorded in (A) VUV APPI and (B) soft X-ray APPI operating in negative ion mode at the probe temperature of 650°C. SLGF dissolved in water was infused from a syringe (10 µL/min) into the Milli-Q water flow (40 µL/min).

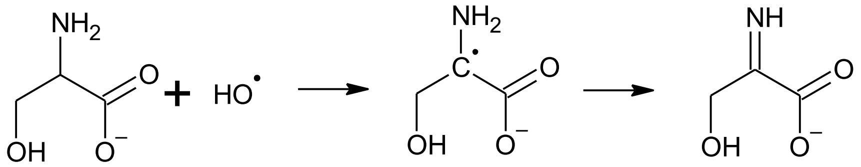

Scheme 1: Reaction of hydroxyl radical with serine as reported in S5.

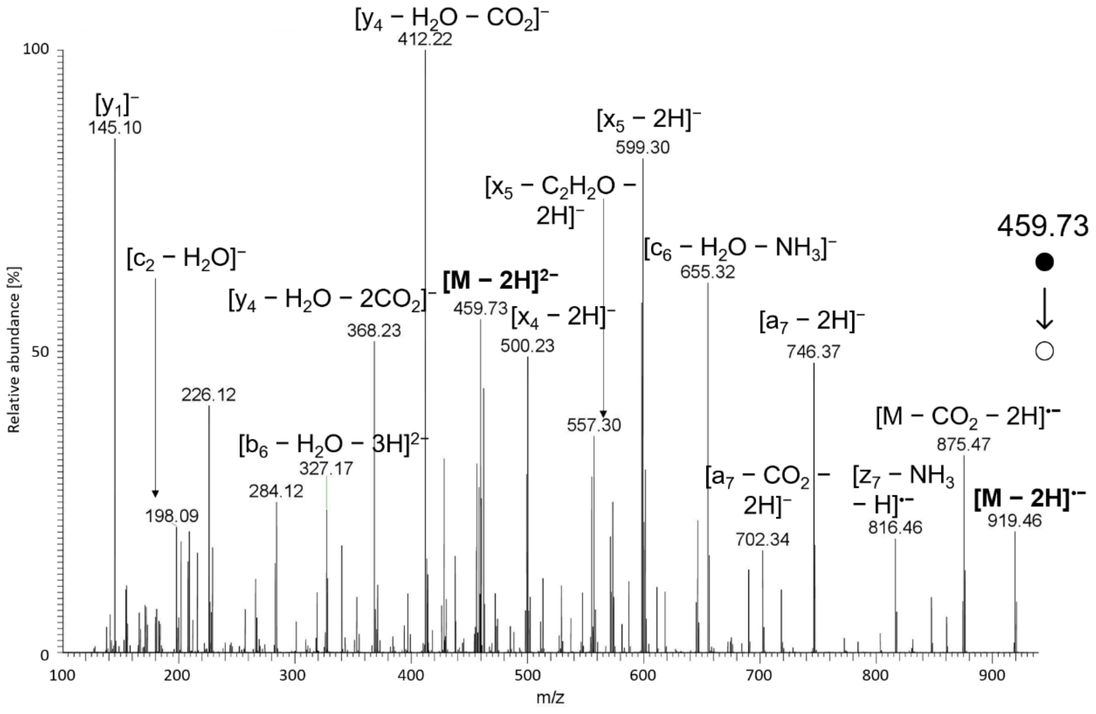

Figure S7. UVPD MS/MS spectrum of [M − 2H]$^{2-}$ for AEFVEVTK. The spectrum was recorded on Orbitrap IQ-X Tribrid equipped with UVPD ion source using 213 nm laser system with 2.5 kHz repetition rate, delivering >1.2 µJ per pulse.

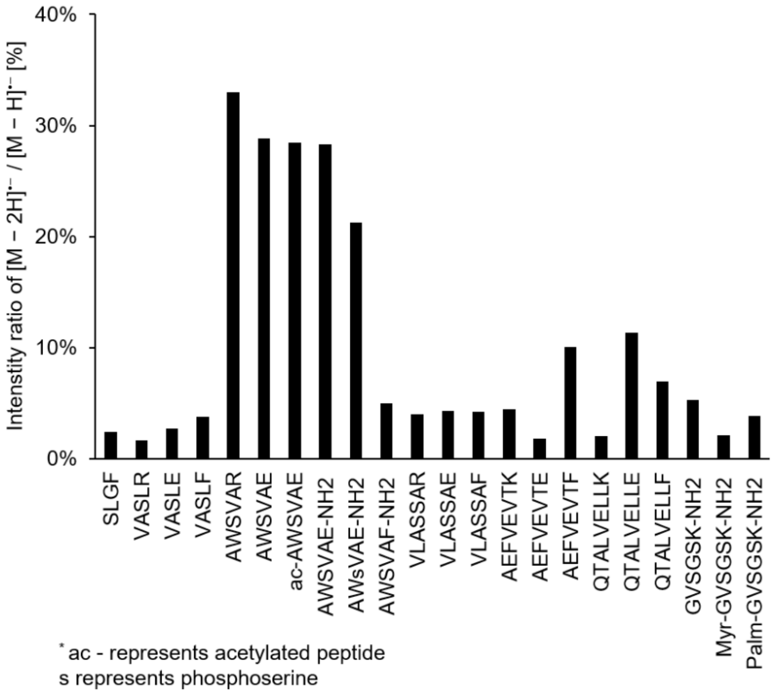

Figure S8. The [M − 2H]•− to [M − H]− intensity ratios calculated from soft X-ray APPI full scan mass spectra. The ion abundances were corrected for the contribution of isobaric ions. The peptides dissolved in water were infused from a syringe (10 µL/min) into the Milli-Q water flow (40 µL/min). The probe temperature was set to 650 °C.

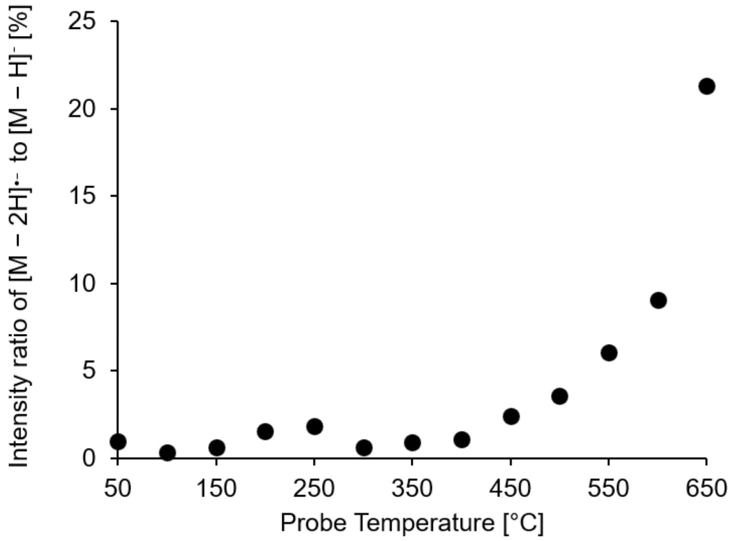

Figure S9. The effect of probe temperature on [M − 2H]•− / [M − H]− intensity ratio in soft X-ray APPI full scan mass spectra. The ion abundances were corrected for the contribution of isobaric ions. AWsVAR-NH_2 dissolved in water was infused from a syringe (10 µL/min) into the Milli-Q water flow (40 µL/min).

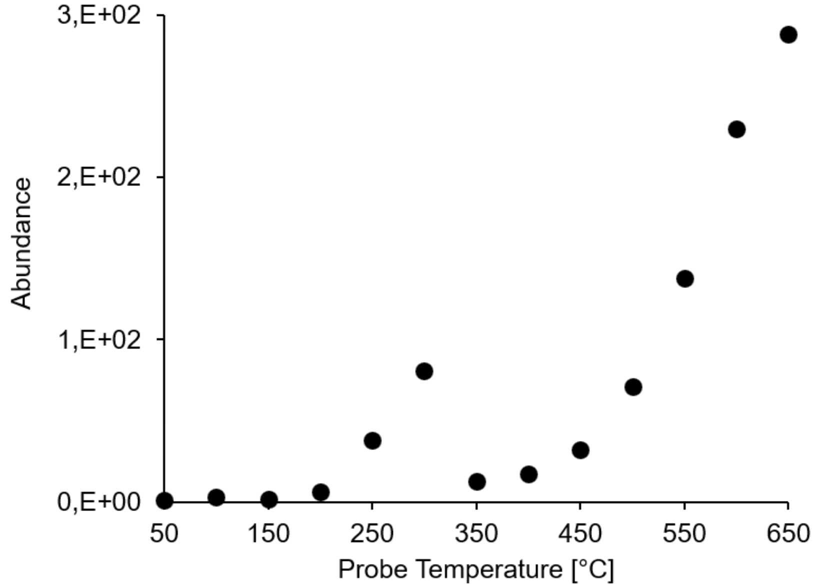

Figure S10. The effect of probe temperature on the abundance of $[M - 2H]^{\bullet-}$ ions in soft X-ray APPI full scan mass spectra. AWsVAR-NH_2 dissolved in water was infused from a syringe (10 µL/min) into the Milli-Q water flow (40 µL/min).

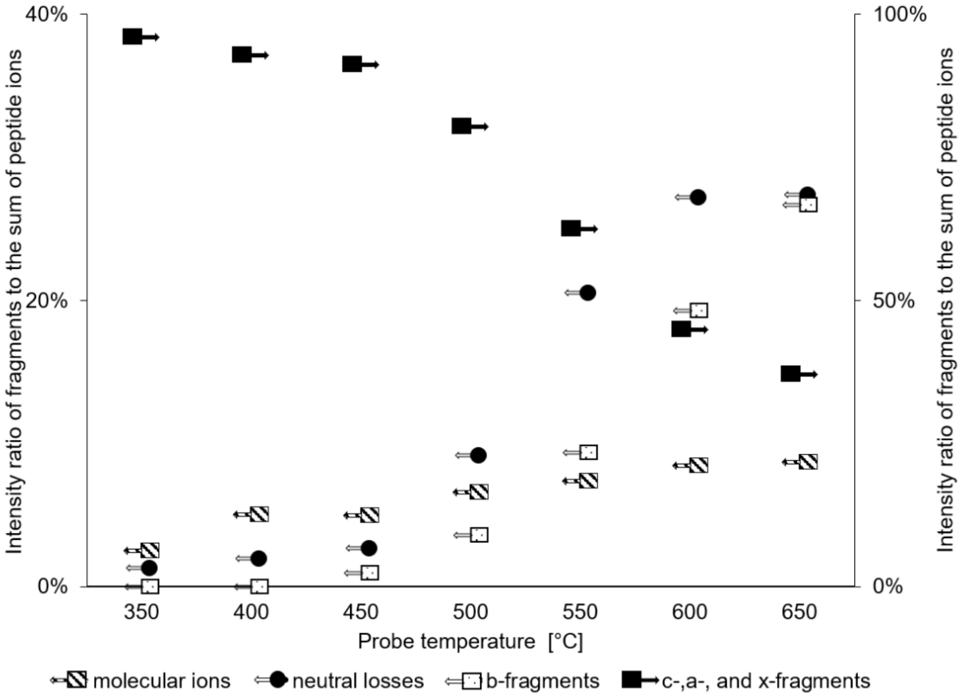

Figure S11. The effect of probe temperature on the intensity ratio of molecular ions ($[M - 2H]^{\bullet-}$, $[M - H]^-$), neutral losses ($[M - NH_3 - H]^-$, $[M - NH{=}C{=}NH - H]^-$, $[M - NH{=}C{=}NH - H_2O - H]^-$), b-fragments and c-, a-, and x- fragments intensity to the sum of peptide ions in soft X-ray APPI. AWSVAR dissolved in water was infused from a syringe (10 µL/min) into the Milli-Q water flow (40 µL/min).

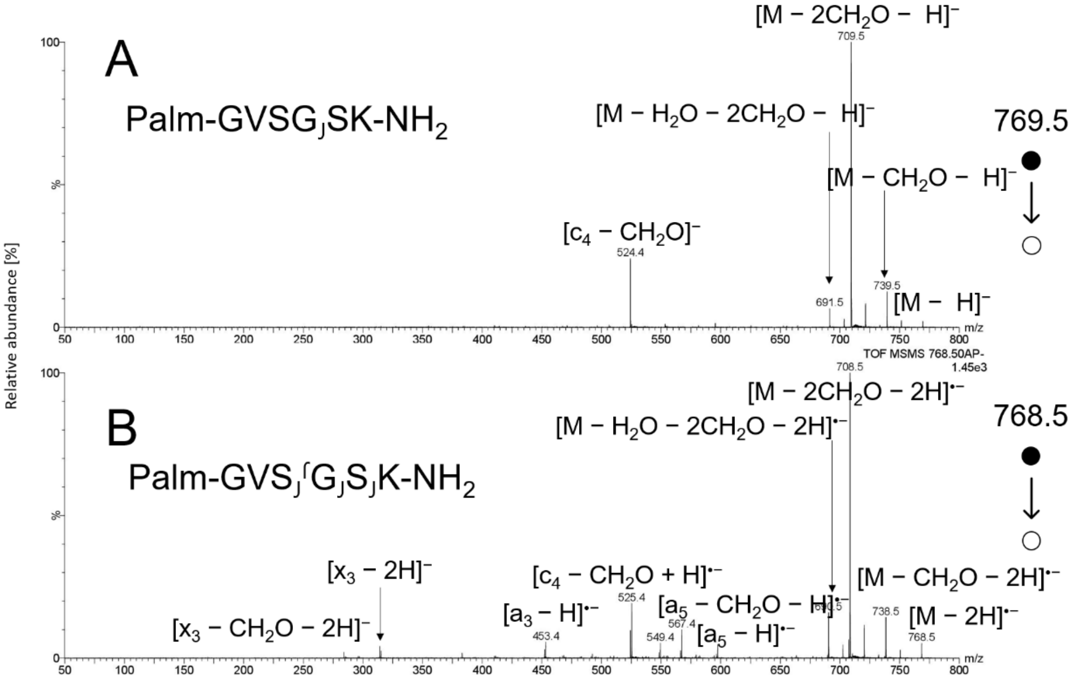

Figure S12. CID MS/MS spectra of (A) [M − H]⁻ (CID 34 V) and (B) [M − 2H]•⁻ (CID 28 V) for N-palmitoyl GVSGSK-NH2.

SI References

(S1) Kostiainen, R.; Kauppila, T. J. *J Chromatogr A* **2009**, *1216* (4), 685-699.
(S2) Robb, D. B.; Blades, M. W. *J Am Soc Mass Spectr* **2005**, *16* (8), 1275-1290.
(S3) Cai, S. S.; Syage, J. A. *J Chromatogr A* **2006**, *1110* (1-2), 15-26.
(S4) Niedermeyer, T. H.; Strohalm, M. *PLoS One* **2012**, *7* (9), e44913.
(S5) LeLacheur, R. M.; Glaze, W. H. *Environ Sci Technol* **1996**, *30* (4), 1072-1080.
